# Atomic scale insights into structure instability and decomposition pathway of methylammonium lead iodide perovskite

**DOI:** 10.1038/s41467-018-07177-y

**Published:** 2018-11-15

**Authors:** Shulin Chen, Xiaowei Zhang, Jinjin Zhao, Ying Zhang, Guoli Kong, Qian Li, Ning Li, Yue Yu, Ningan Xu, Jingmin Zhang, Kaihui Liu, Qing Zhao, Jian Cao, Jicai Feng, Xinzheng Li, Junlei Qi, Dapeng Yu, Jiangyu Li, Peng Gao

**Affiliations:** 1 Electron Microscopy Laboratory, School of Physics, Peking University, Beijing, 100871 China; 2 State Key Laboratory of Advanced Welding and Joining, Harbin Institute of Technology, Harbin, 150001 China; 3 International Center for Quantum Materials, School of Physics, Peking University, Beijing, 100871 China; 4 School of Materials Science and Engineering, Shijiazhuang Tiedao University, Shijiazhuang, 050043 China; 5 X-ray Science Division, Argonne National Laboratory, Lemont, IL 60439 USA; 6 Center for Nanochemistry, College of Chemistry and Molecular Engineering, Peking University, Beijing, 100871 China; 7 Oxford Instruments Technology (Shanghai) Co. Ltd., Shanghai, 200233 China; 8 State Key Laboratory for Mesoscopic Physics, School of Physics, Peking University, Beijing, 100871 China; 9 Collaborative Innovation Center of Quantum Matter, Beijing, 100871 China; 10 Department of Physics, South University of Science and Technology of China, Shenzhen, 518055 China; 11 Shenzhen Key Laboratory of Nanobiomechanics, Shenzhen Institutes of Advanced Technology, Chinese Academy of Sciences, Shenzhen, 518055 Guangdong China; 12 Department of Mechanical Engineering, University of Washington, Seattle, WA 98195-2600 USA

## Abstract

Organic–inorganic hybrid perovskites are promising candidates for the next-generation solar cells. Many efforts have been made to study their structures in the search for a better mechanistic understanding to guide the materials optimization. Here, we investigate the structure instability of the single-crystalline CH_3_NH_3_PbI_3_ (MAPbI_3_) film by using transmission electron microscopy. We find that MAPbI_3_ is very sensitive to the electron beam illumination and rapidly decomposes into the hexagonal PbI_2_. We propose a decomposition pathway, initiated with the loss of iodine ions, resulting in eventual collapse of perovskite structure and its decomposition into PbI_2_. These findings impose important question on the interpretation of experimental data based on electron diffraction and highlight the need to circumvent material decomposition in future electron microscopy studies. The structural evolution during decomposition process also sheds light on the structure instability of organic–inorganic hybrid perovskites in solar cell applications.

## Abstract (web summary)

Hybrid perovskites are highly promising for photovoltaic applications, but they are prone to decomposition. Here, the authors probe the stability of CH_3_NH_3_PbI_3_ films in a transmission electron microscope, defining the threshold conditions to avoid damage under the electron beam, and describing a decomposition pathway.

## Introduction

Solar cells based on organic–inorganic hybrid perovskites (CH_3_NH_3_PbI_3_, MAPbI_3_) have attracted widespread research attention due to their low synthesis cost and high-power conversion efficiency (PCE)^1–3^. Despite the great progress in increasing the device PCE from initial 3.8%^4^ to the most recent 23.3%^5^ in just one decade, the commercialization of this technology remains hindered by the stability issues of materials. It has been reported that the perovskites rapidly degrade under increased temperature^6^, oxygen^7^, moisture^8^, and UV light illumination^9^, often due to the structure instability caused by electromigrations^10^, ion migration,^11^ and interfacial relationships^12^. In fact, with increasing temperature, visible diffusion of iodine initiates at a temperature below 150 °C and lead migration is induced at a higher temperature around 175 °C, causing the degradation of perovskite and formation of PbI_2_^6^. The ion migration induced by the electric field, heat, and light illumination also contributes to the hysteresis in J–V curves^13,14^, which in turn leads to poor long-term stability resulted from significant structural changes, including the lattice distortion and the phase decomposition. Thus, it is essential to characterize the intrinsic structure as well as structure stability and the decomposition pathway of these materials to improve organic–inorganic hybrid perovskites, which motivates our present transmission electron microscopy (TEM) study on perovskite MAPbI_3_.

Although TEM has been widely used for structure characterization of perovskite MAPbI_3_^15–17^, the atomically resolved imaging remains elusive due to its electron beam-sensitive nature^18^. Thus, the electron diffraction (ED) or fast Fourier transform (FFT) pattern based on lattice fringes becomes a common preference to identify the phase of perovskite. However, in most of the experiments, the observed ED or FFT patterns do not really match those of a perfect tetragonal perovskite phase exactly^16,17,19–23^^,^. For example, {110} diffraction spots along the [001] direction are usually not observed in electron microscopy even though these reflections are visible from X-ray diffraction (XRD) measurements^24–29^. Long et al.^24^ identified a textured thin MAPbI_3_ film to be a tetragonal [001] zone axis even though {110} spots were not observed. Park et al.^25^ prepared a cross-sectional whole device of glass/FTO/mp-TiO_2_/MAPbI_3_/spiro-OMeTAD, and similarly the {110} spots were absent. Polarz et al.^26^ developed metal–organic porous single-crystalline MAPbI_3_ and also did not observe the (1$\bar 1$0) spots. Furthermore, Tang et al.^27^, Vela et al.^28^, and Zhu et al.^29^ found that the {110} spots were missing in MAPbI_3_ nanowires. Segawa^30^ explained that the (110) and (1$\bar 1$0) diffraction spots are inherently difficult to be detected experimentally due to the low ED intensity of organic–inorganic halide compounds. Despite the absence of some expected diffraction spots, the corresponding structures were often identified as a tetragonal perovskite phase, as summarized in Supplementary Table 1. Nevertheless, {110} diffraction spots of perovskite^31^ have been captured from a rapid acquisition with the dose rate as low as 1 e Å^−2 ^s^−1^, suggesting that the absence of {110} reflections of perovskite may be related to structure degradation during electron microscopy characterizations. From an energetic point of view, electron beam illumination, optical illumination, heating, and the electric field are similar, enabling ions to overcome the migration barriers and thus inducing the structure transition^32–34^. Revealing the structure evolution of MAPbI_3_ under electron illumination, especially its decomposition pathway, therefore, can help us to understand material degradation in practical solar cell devices.

In this paper, we use atomically resolved Z-contrast imaging, diffraction analysis, and quantitative energy- dispersive X-ray spectroscopy (EDX) in an aberration-corrected TEM in an attempt to understand the structure and structure evolution of single-crystalline tetragonal MAPbI_3_. We found that the perovskite structure undergoes complex phase changes under the electron beam illumination. The decomposition of MAPbI_3_ is likely initiated with the loss of I ions to subsequently form superstructure MAPbI_2.5_, followed by the loss of MA and additional I ions to form MA_*y*_PbI_2.5–*z*_ (0 ≤ *y* ≤ 1 and 0 ≤ *z* ≤ 0.5) accompanied with the disappearance of a superstructure, leading to its final decomposition into PbI_2_. During this process, the ED patterns change subtly that is often overlooked, and thus the evolving and decomposed phases are often incorrectly identified as perovskite. Indeed, if we can only observe {2h, 2k, 0} diffraction spots along the [001] direction and while the {2h+1, 2k+1, 0} reflections [e.g., (110)] are absent, it is highly likely that the tetragonal perovskite phase has already decomposed into a hexagonal PbI_2_ structure along the [$\bar 4$41] zone axis. We also identify the critical electron dose limit to obtain the intrinsic perovskite structure. Our findings impose important questions on some earlier experimental interpretations of ED measurements of MAPbI_3_ and highlight the need to circumvent material degrading in future experiments. The identified optimal TEM conditions to obtain the intrinsic perovskite structure can be used to guide the future electron microscopy characterization of these materials. The understanding of the degradation process also sheds light into the general stability issue of the organic–inorganic hybrid perovskites in solar cell applications.

## Results

### Mismatches in ED patterns

**Fig. 1:**
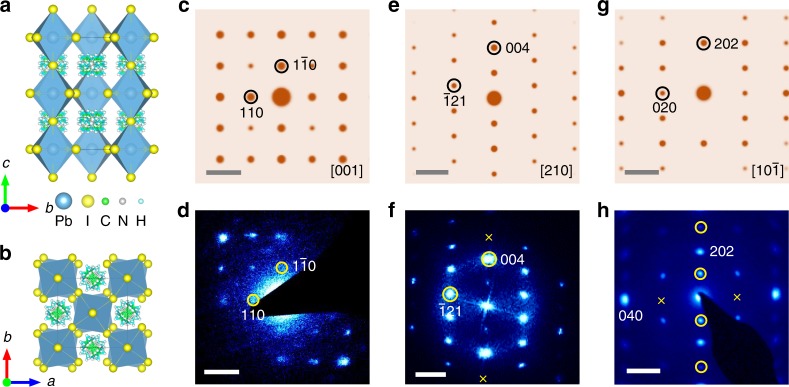
The atomic structure of tetragonal MAPbI_3_. The atomic models of tetragonal MAPbI_3_ along **a** [100] and **b** [001] zone axis. The simulated electron diffraction (ED) patterns of tetragonal MAPbI_3_ along **c** [001], **e** [210], and **g** [10$\bar 1$] zone axes, respectively. **d**, **h** The selected area electron diffraction (SAED) and **f** fast Fourier transform (FFT) pattern of tetragonal MAPbI_3_. Note that there is a subtle difference between the experiment and simulation. The {110} diffraction spots appear to be dimmer in **d**, and the (00$2$) spot almost vanishes and the (006) spot disappears marked by yellow crosses in **f**, and the (020) spot is not observed marked by yellow crosses but additional {2h+1, 0, 2h+1} reflections [e.g., (101)] appear indicated by yellow circles in **h**. Scale bar, 2 nm^−1^ (**c**–**h**)

MAPbI_3_ crystals were self-grown on FTO/TiO_2_ substrates, as reported in our previous study^35,36^. The powder XRD (Supplementary Fig. 1a) indicates a good crystallinity of tetragonal MAPbI_3_^37,38^. The single-crystalline nature is further confirmed by synchrotron X-ray diffraction (XRD) (Supplementary Fig. 1b) with the measured (004)_c_ reflection denoted in the pseudo-cubic setting. However, when MAPbI_3_ crystal was put into TEM, the acquired ED and FFT patterns do not match those of a perfect tetragonal perovskite structure. The atomistic models of tetragonal MAPbI_3_ along [100] and [001] axis are shown in Fig. 1a, b, and the corresponding ED patterns simulated from the perfect structure are shown in Fig. 1c, e, and g. A subtle difference between the experimental SAED (Fig. 1d and h) and FFT pattern (Fig. 1f) acquired from regions in Supplementary Fig. 2 and the simulated ones can be noted after careful comparison. For example, Fig. 1d is identified to be the [001] zone axis of tetragonal MAPbI_3_, which is similar to the simulated ED pattern in Fig. 1c, yet {110} diffraction spots do become dimmer. More importantly, Fig. 1f is identified to be the [210] zone axis, wherein the (002) diffraction spot can hardly be seen. Figure 1h is identified to be the [10$\bar 1$] zone axis, wherein the (020) spot is not observed while an additional (101) spot appears. Therefore, whether these SAED patterns indeed correspond to tetragonal MAPbI_3_ is questionable and needs further investigation.

### Atomic structure of PbI_2_

**Fig. 2:**
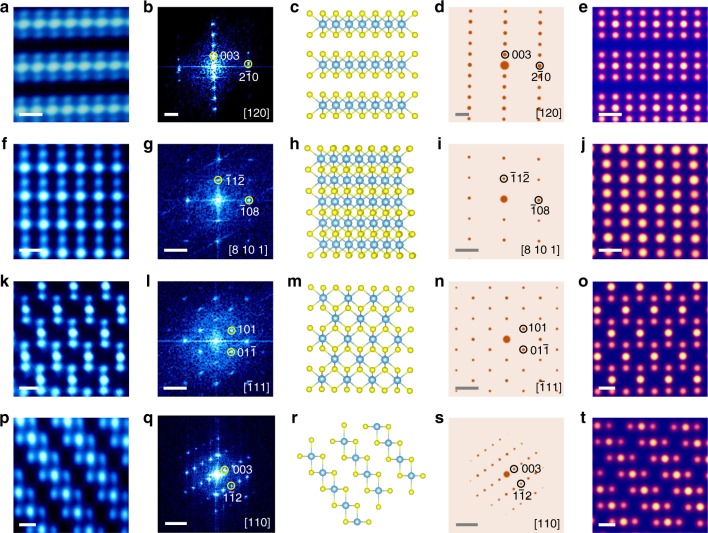
The atomic structure of PbI_2_ along different zone axes. **a**, **f**, **k**, **p** STEM images of PbI_2_ along [120], [8 10 1], [$\bar 1$11], and [110] zone axes with **b**, **g**, **l**, **q** the corresponding FFT patterns, **c**, **h**, **m**, **r** atomic ball-and-stick models, **d**, **i**, **n**, **s** simulated ED patterns, and **e**, **j**, **o**, **t** simulated STEM images. Scale bar, 0.3 nm (**a**, **e**, **f**, **j**, **k**, **o**, **p**, **t**); 2 nm^−1^ (**b**, **d**); 3 nm^−1^ (**g**, **i**, **l**, **n**, **q**, **s**)

To resolve the subtle difference between experimental and simulated ED patterns, atomically resolved Z-contrast images were recorded at 80 kV. An atomically resolved STEM image in Fig. 2a shows stripes with a measured distance of 0.72 nm. Based on the corresponding FFT pattern (Fig. 2b), the viewing direction is often mistaken as the [10$\bar 1$] of MAPbI_3_ without noticing that additional (101) reflection appears while the (020) spot is missing. After careful analysis, we found that these stripes are in fact not from MAPbI_3_ but PbI_2_, which can be illustrated by the ball-and-stick model of the PbI_2_ (Fig. 2c). Furthermore, simulated HAADF-STEM (Fig. 2e) was carried out to compare with the experimental image contrast (Fig. 2a), which shows good agreement. The experimental FFT pattern (Fig. 2b) can be identified to be the [120] direction of PbI_2_, which matches the simulated ED pattern (Fig. 2d) well. The detailed atomistic configurations of the hexagonal PbI_2_ (space group: R-3m:H, *a* = *b* = 0.4557 nm, *c* = 2.0937 nm, *α* = *β* = 90°, and *γ* = 120°) are shown in Supplementary Fig. 3^39^, wherein the layered PbI_2_ structure is composed of Pb–I octahedra. The stability of this structure at room temperature is further confirmed by the molecular dynamics (MD) simulation at 300 K.

Additional atomic structure data along different directions (Fig. 2f–t) further confirm that the original sample of MAPbI_3_ is indeed decomposed into PbI_2_. These STEM images have been filtered and the pristine images are shown in Supplementary Fig. 4. Figure 2f shows the alternating Pb and I arrangements of PbI_2_, illustrated by the ball-and-stick model in Fig. 2h. The FFT pattern (Fig. 2g) from the region (Supplementary Fig. 4b) is identified to be the [8 10 1] zone axis, which is consistent with simulated ED pattern (Fig. 2i). In addition, the simulated STEM image in Fig. 2j also shows a similar contrast, compared with Fig. 2f. Likewise, atomic STEM images along [$\bar 1$11] and [110] zone axes have also been analyzed as shown in Fig. 2k–t. These STEM images well match the corresponding ball-and-stick models and simulated STEM images of the PbI_2_, and the corresponding FFT patterns are also consistent with the simulated ED patterns of the PbI_2_. Additional data of PbI_2_ are presented in Supplementary Fig. 5, showing a subtle difference with simulated MAPbI_3_ in the ED pattern in Supplementary Fig. 6. It is worth noting that both ED patterns of [8 10 1] and [$\bar 1$11] zone axes of PbI_2_ are often easily mistaken for the [10$\bar 1$] direction of MAPbI_3_ when the missing of (020) diffraction spot from MAPbI_3_ is not recognized.

### Structural comparison between MAPbI_3_ and PbI_2_

**Fig. 3:**
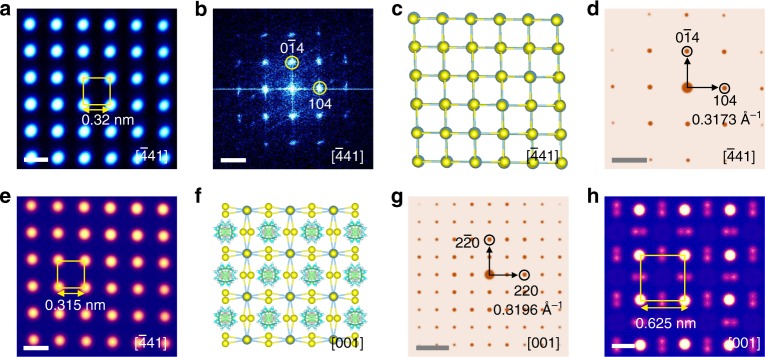
Structure and ED difference between PbI_2_ and tetragonal MAPbI_3_. **a** A STEM image of PbI_2_ viewing along the [$\bar 4$41] direction, judged from **b** the corresponding FFT pattern. **c** The atomic ball-and-stick model, **d** the simulated ED pattern, and **e** simulated STEM image of PbI_2_ along the [$\bar 4$41] zone axis. **f** The atomic ball-and-stick model, **g** the simulated ED pattern, and **h** simulated STEM image of MAPbI_3_ along the [001] zone axis. The ED patterns of PbI_2_ and MAPbI_3_ are similar with a slight difference that the {2h+1, 2h+1, 0} reflections [e.g., (110)] of MAPbI_3_ are not observed in that of PbI_2_. Besides, the distance between two brighter atoms is 0.625 nm for MAPbI_3_, which is about twice the distance between two brighter atoms (0.315 nm) of PbI_2_. Scale bar, 0.3 nm (**a**, **e**, **h**); 3 nm^−1^ (**b**, **d**, **g**)

It is quite revealing to highlight the difference in atomic structures and ED patterns between MAPbI_3_ and PbI_2_. Figure 3a is a HAADF-STEM image of PbI_2_ viewing along the [$\bar 4$41] direction judging from the corresponding FFT pattern (Fig. 3b) as well as the SAED and TEM images (Supplementary Fig. 7). The atomistic model, simulated ED pattern, and simulated STEM image of PbI_2_ along this zone axis are shown in Fig. 3c–e. However, in many previous studies^16,17,19–29^, this ED or FFT pattern has been mistakenly identified as [001] zone axis of tetragonal MAPbI_3_ without noticing the absence of {110} diffraction spots (Supplementary Table 1). For comparison, based on the atomistic model in Fig. 3f, we also carried out the simulations of ED (Fig. 3g) and HAADF image (Fig. 3h) for MAPbI_3_ along the [001] zone axis. Note that the distance in reciprocal space for (220) reflection of tetragonal MAPbI_3_ is 0.3196 Å^−1^ (Fig. 3g), which is extremely close to that of (104) and (0$\bar 1$4) reflections (0.3173 Å^−1^) for PbI_2_ (Fig. 3d), making it difficult to distinguish them only based on the SAED pattern. Despite of the similarity in their ED patterns, the simulated atomic structures of these two phases are quite different. For tetragonal MAPbI_3_ along the [001] zone axis, the contrast of atom columns is not uniform (Fig. 3h), in comparison with the uniform contrast in PbI_2_ along [$\bar 4$41] in Fig. 3e. Moreover, the distance between two brighter atom columns is 0.625 nm for MAPbI_3_ (Fig. 3h), which is twice the distance between two brighter atom columns (0.315 nm) of PbI_2_ (Fig. 3e).

### The structure evolution during decomposition

**Fig. 4:**
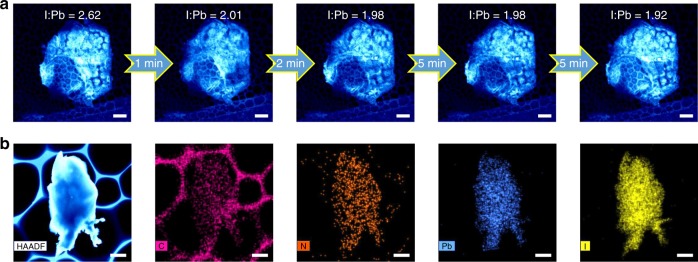
The quantitative elemental analysis during the degradation of MAPbI_3_. **a** Scanning electron microscopy (SEM) images with the atomic ratio for I/Pb acquired from the corresponding energy-dispersive X-ray spectroscopy (EDX) mappings in Supplementary Fig. 10, showing that the atomic ratio of I/Pb gradually decreases to 2. The beam was blank for a certain time indicated by the arrows after every spectrum was acquired. **b** A STEM image and the corresponding EDX mappings for the quantitative elemental analysis (Supplementary Table 2). Scale bar, 5 μm (**a**); 500 nm (**b**)

The evolution of tetragonal MAPbI_3_ decomposing into hexagonal PbI_2_ is important for understanding the material degradation, and it can be captured at a low magnification with SEM and a series of EDX mappings (Fig. 4). During the process, the I/Pb atom ratio decreases from the initial 2.62 to the final 1.92 due to the volatilization of the iodine^40,41^ (Supplementary Fig. 8), consistent with the formation of PbI_2_. The quantitative EDX mapping in the STEM mode at 80 kV also shows that the average atom ratio for I/Pb is close to 2 (Supplementary Table 2, based on the spectrum in Supplementary Fig. 9). Additional STEM-EDX mapping was conducted on other samples (Supplementary Fig. 10) showing similar observations.

**Fig. 5:**
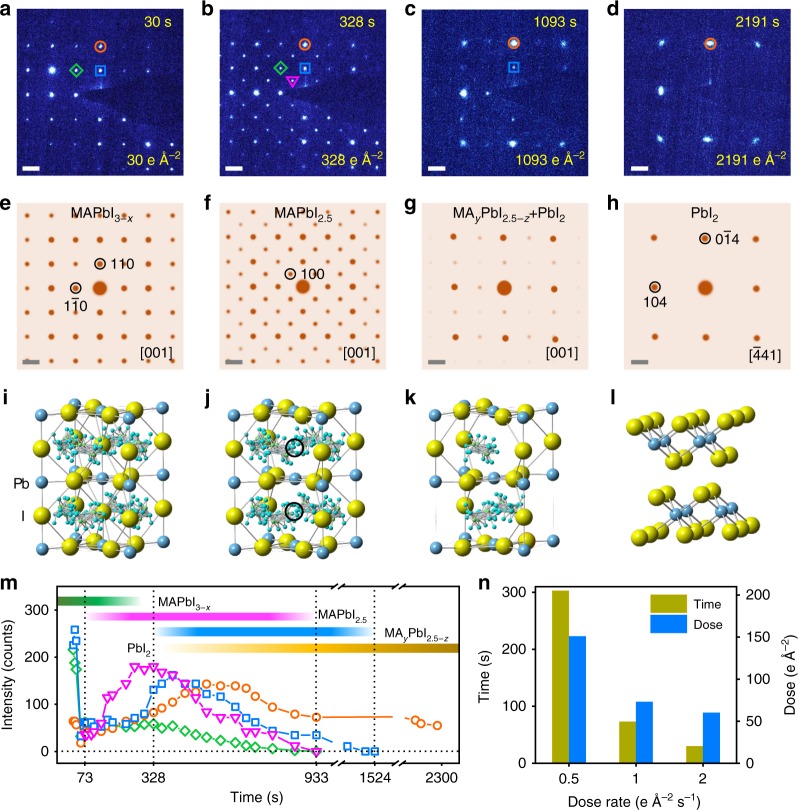
The structural evolution during the decomposition from MAPbI_3_ to PbI_2_. **a**–**d** Time-series SAED patterns showing that the structure decomposes from **a** MAPbI_3–*x*_ [001] zone axis (0 < *x* < 0.5), to **b** intermediate phase MAPbI_2.5_, **c** MA_*y*_PbI_2.5–*z*_ (0 ≤ *y* ≤ 1 and 0 ≤ *z* ≤ 0.5), and **d** PbI_2_ [$\bar 4$41] zone axis sequentially under a dose rate of 1 e Å^−2^ s^−1^. The cumulative electron dose and time are labeled in each pattern. The blue squares mark the (110) reflection of MAPbI_3_ in **a**, MAPbI_2.5_ in **b**, and MAPbI_2.5_ and MA_*y*_PbI_2.5–*z*_ in **c**. The green diamonds mark the (200) reflection of MAPbI_3_ in **a**, and MAPbI_2.5_ in **b**. The orange circles mark the (220) reflection of MAPbI_3_ in **a**, MAPbI_2.5_ in **b**, MAPbI_2.5_ and MA_*y*_PbI_2.5–*z*_ in **c**, and ($0\bar 1$4) of PbI_2_ in **d**. The pink triangles mark the (100) reflection of MAPbI_2.5_ in **b**. **e**–**h** The corresponding simulated ED patterns of **e** MAPbI_3_, **f** MAPbI_2.5_, **g** 35% MA_*y*_PbI_2.5–*z* _+ 65% PbI_2_ (see Supplementary Fig. 12), and **h** PbI_2_. Note that MA_*y*_PbI_2.5–*z*_ (0 ≤ *y* ≤ 1 and 0 ≤ *z* ≤ 0.5) with random vacancies should have the same ED pattern with MA_*y*_PbI_2.5–*z*_ (*y* = 0 and *z* = 0.5) without considering the very diffused reflections from random vacancies. **i**–**l** The atomistic structures of **i** MAPbI_3_, **j** MAPbI_2.5_, **k** MA_*y*_PbI_2.5–*z*_ (0 ≤ *y* ≤ 1 and 0 ≤ *z* ≤ 0.5), and **l** PbI_2_. The black circles in **j** represent the ordered iodine vacancies. **m** The diffraction intensity is plotted as a function of time (total dose). The pink triangles, orange circles, blue squares, and green diamonds correspond to those label shapes in **a**–**d**. The green, pink, blue, and orange color bars roughly estimate the existence of MAPbI_3–*x*_, MAPbI_2.5_, MA_*y*_PbI_2.5–*z*_, and PbI_2_ at different stages. Note that the values of the intensities for (110) (blue squares) and (100) (pink triangles) spots are set to be 1.8 and 6 times of the original values for comparison. **n** During the decomposition of the perovskite at different dose rates, the measured critical electron dose and time for the appearance of a superstructure. Scale bar, 1 nm^−1^ (**a**–**h**)

In order to capture the intrinsic perovskite structure and investigate the detailed degradation process, the evolution of ED patterns under a low-dose rate ~1 e Å^−2^ s^−1^ is recorded in Fig. 5a–d viewing along [001] MA_*y*_PbI_3–*x*_ (0 ≤ *y* ≤ 1 and 0 ≤ *x* ≤ 1) (or $[\bar 441]$ of PbI_2_). These time-series SAED patterns match the corresponding simulated ones (Fig. 5e–h) based on the atomistic structures (Fig. 5i–l). During the decomposition, we observe an intermediate phase with superstructure spots (Fig. 5b), which can be fitted by MAPbI_2.5_ with ordered iodine vacancies as shown in Fig. 5j. Compared with the pristine MAPbI_3_ in Fig. 5i, part of iodine ions on the (001) plane indicated by the black circles in Fig. 5j are lost to form the superstructure MAPbI_2.5_. The corresponding simulated ED pattern of MAPbI_2.5_ in Fig. 5f matches well with the experimental result in Fig. 5b. Furthermore, from another viewing direction of [100] of perovskite, the ED pattern corresponding to superstructure MAPbI_2.5_ is also observed (see details in Supplementary Fig. 11). In fact, iodine ions are the most likely mobile ions due to the lower diffuse barrier in MAPbI_3_^11,42,43^, similar to the oxygen ions in the oxide perovskites^44^, and the migration of iodine ions in MAPbI_3_ indeed has been reported from the previous studies^10,14,45^. Thus, we infer that the superstructure is likely caused by the formation of ordered I vacancies. Moreover, forming such an intermediate superstructure MAPbI_2.5_ tends to minimize the repulsive interaction between iodine ions as well as the elastic coherence strain energy, similar to the staging phases in the lithium ion battery materials^46,47^.

With further increased electron beam dose, the structure continues to lose MA and additional I ions to form MA_*y*_PbI_2.5–*z*_ (0 ≤ *y* ≤ 1 and 0 ≤ *z* ≤ 0.5) (Fig. 5k), which is accompanied by the disappearance of the superstructure. Finally, the perovskite structure framework collapses to form the layered PbI_2_ structure (Fig. 5l). Note that the ED pattern in Fig. 5c can be regarded as a combination of MA_*y*_PbI_2.5–*z*_ along [001] and PbI_2_ along [$\bar 4$41] directions, consistent with the overlap of the corresponding simulated ED patterns in Fig. 5g (see details in Supplementary Fig. 12). It is also interesting to note that the previous study has proposed the reaction pathway from PbI_2_ to MAPbI_3_ during synthesis^48^, and the layered structure of MA_1+*x*_PbI_3+*y*_ was proposed as the intermediate phase, similar to our observation of MA_*y*_PbI_2.5–*z*_.

To better understand the decomposition process from MAPbI_3_ to PbI_2_, we also investigate the intensity changes of diffraction spots during the decomposition as shown in Fig. 5m. Under the electron beam illumination, the intensities of the MAPbI_3_ (220), (110), and (200) spots decrease, indicating that the perovskite structure becomes defective. At 73 s, the (100) superstructure reflection starts to appear likely due to the formation of MAPbI_2.5_. With the growing of MAPbI_2.5_, its (100) reflection intensity gradually increases. After a peak intensity of MAPbI_2.5_ (100) at 328 s, MAPbI_2.5_ further loses I and MA to form MA_*y*_PbI_2.5–*z*_ (0 ≤ *y* ≤ 1 and 0 ≤ *z* ≤ 0.5), leading to the reduction of superstructure reflection intensity. Meanwhile, the perovskite structure collapses and decomposes into a layered PbI_2_ structure. After 933 s, the superstructure MAPbI_2.5_ has completely disappeared. And after 1524 s, the reflections of MA_*y*_PbI_2.5–*z*_ (0 ≤ *y* ≤ 1 and 0 ≤ *z* ≤ 0.5) phase disappear and the intensity of the (0$\bar 1$4) spot of PbI_2_ is stabilized, indicating that the structure has completely decomposed into PbI_2_.

Finally, we investigate the effect of the dose rate on the degradation, and we determine the critical electron dose limit to obtain the intrinsic perovskite structure. Note that the perovskite framework remains until the MA_*y*_PbI_2.5–*z*_ (0 ≤ *y* ≤ 1 and 0 ≤ *z* ≤ 0.5) collapses. Therefore, it is reasonable to expect that before the formation of MAPbI_2.5_ superstructure, the perovskite structure with a low density of iodine vacancies is robust and close to the ideal pristine structure. We find that typically the formation of MAPbI_2.5_ starts at 303 s under a dose rate of 0.5 e Å^−2^ s^−1^ (total dose: 151 e Å^−2^) and 73 s under a dose rate of 1 e Å^−2^ s^−1^ (total dose: 73 e Å^−2^), while at 2 and 4 e Å^−2^ s^−1^, the decomposition has already begun at the first SAED images (~30 s of exposure) (see details in Supplementary Fig. 13). Note that the decomposition not only depends on the total dose, but is also sensitive to dose rate, with higher rates resulting in accelerated decomposition under a similar total dose.

## Discussion

The MAPbI_3_ is extremely sensitive to electron irradiation and thus it is easy to decompose into PbI_2_ accompanied by subtle changes in ED, i.e., some of the diffraction spots are missing or weaken, or additional spots appear. Particularly, if we can only observe {2h, 2k, 0} diffraction spots along the [001] direction while the {2h+1, 2k+1, 0} reflections [e.g., (110)] are absent, it is highly likely that the perovskite structure has already decomposed into PbI_2_. However, many previous studies ignored such subtle changes and incorrectly identified the decomposed PbI_2_ to be MAPbI_3_ based on the ED or FFT analysis. Consequently, extra attention must be paid during analyzing the electron microscopy data for these materials.

More importantly, we find that the decomposition of MAPbI_3_ is likely initiated with the loss of I ions to subsequently form superstructure MAPbI_2.5_, followed by the loss of MA and additional I ions to form MA_*y*_PbI_2.5–*z*_ (0 ≤ *y* ≤ 1 and 0 ≤ *z* ≤ 0.5) accompanied by the disappearance of a superstructure, and finally decomposes into PbI_2_. Typically, under electron beam illumination with a dose rate of 0.5 e Å^−2^ s^−1^, the MAPbI_3_ starts to change into MAPbI_2.5_ with ordered I vacancies after 303 s, leaving sufficient time to acquire the ED for an intrinsic perovskite structure, while a higher dose rate results in faster decomposition under a similar total dose. With a dose rate higher than 2 e Å^−2^ s^−1^, decomposition has already begun at the first SAED images. These identified TEM conditions can be used to guide future electron microscopy characterization of these materials.

Moreover, considering the similarities between electron beam illumination and optical illumination, as well as the heating and electric field, during which the ions gain energy to overcome the migration barriers to trigger the phase transitions, we believe that the electro-driven ion migration and heating will likely lead to a similar structural degradation process, which may be responsible for the poor long-term device stability. In this sense, the observed decomposition of perovskite under the electron beam illumination can help us to understand the structure instability issue for these materials under device operation, which is currently under further investigation.

## Methods

### MAPbI_3_ synthesis

The single-crystal perovskite MAPbI_3_ was synthesized as follows. Two FTO/TiO_2_ substrates were clamped together using the fixed-size card slots. In total, 2 mL of precursor solution of PbI_2_/MAI (molar ratio 1:1) was prepared in γ-butyrolactone. After 12 h of continuous stirring, a homogeneous MAPbI_3_ solution at a concentration of 1.3 mol/L was obtained, and then filtered using a polytetrafluoroethylene (PTFE) filter with 0.22-µm pore size. The clamped FTO/TiO_2_ substrates were then vertically dipped into a 10-mL beaker containing MAPbI_3_ precursor solution kept on a hot plate at 120 ^o^C in nitrogen atmosphere, and the feeding precursor solution was added every 12 h. After a certain period of 27 days, the substrates with a self-grown film were taken out, and then dried at 120 ^o^C for 10 min in nitrogen glovebox.

### Data acquisition and analysis

Powder XRD patterns were obtained on D8 Advance diffractometer using Cu Kα radiation (40 kV and 40 mA) with a scanning rate of 4° min^−1^ for wide-angle test increment. The morphologies of the sample were examined by SEM (ZEISS Gemini SEM 300), and the SEM-EDX was carried out at 10 kV with 1 min for acquiring the spectra, using a current of ~ 1 nA. The beam was kept blank for several minutes after each spectrum was acquired because the sample is sensitive to the electron beam.

Single-crystal X-ray diffraction was carried out at Sector 7-ID-C, Advanced Photon Source, Argonne National Laboratory, using 10 -keV X-ray with a 300 × 300 μm^2^ beam size as defined by a slit. A Huber six-circle diffractometer coupled with a PILATUS 100-K area detector was employed for measurements of the single-crystalline samples. The detector was placed downstream from the samples such that ~ 8° coverage in the 2*θ*-angle and ~ 3° in the *χ*-angle was obtained. Rocking (*ω*-angle) scans around each reflection were recorded and for the data presented, the resultant 3D data volume was reduced by integrating the rocking angles.

High-resolution TEM, HAADF STEM images were acquired at an aberration-corrected FEI (Titan Cubed Themis G2) operated at 80 kV equipped with an X-FEG gun and Bruker Super-X EDX detectors. STEM images were acquired with a beam current of 6–10 pA, a convergence semi-angle of 25 mrad, and a collection semi-angle snap in the range of 53–260 mrad. The STEM-EDX mapping was obtained with a beam current of 1 nA and counts ranging from 2k cps to 8k cps for ~ 5 min. The SAED patterns for Fig. 5, Supplementary Fig. 11, and Fig. 13 were acquired at 300 kV.

Under the STEM mode for Figs. 2 and 3, the dose rate is estimated to be 220–360 e Å^−2^ s^−1^ which is calculated by dividing the screen current (6 ~ 10 pA) by the area of the raster^49^. To get a satisfactory STEM image, we need to carefully align the zone axis and adjust the astigmatism and the focus before image acquisition, which is time-consuming and costs about 5–10 min. To record the SAED patterns of Fig. 1d, the sample is always observed at low magnifications (10000–30000) and the dose rate is estimated to be 100–300 e Å^−2^ s^−1^. It usually takes 30–100 s to record a SAED image after the sample comes into sight. Under these conditions, judging from the data we acquired, the samples were decomposed due to large electron doses. To obtain the ideal pristine structure, we make the dose rate as low as possible. For Fig. 5, Supplementary Figs. 11 and 13, the dose rate ranges from 0.5 to 4 e Å^−2^ s^−1^.

The simulations of the ED pattern were performed by using Crystalmaker software, and the ball-and-stick models were formed using Vesta software. The FFT and inverse FFT patterns were obtained using DigitalMicrograph (Gatan) software. HAADF images in Fig. 2, Fig. 3, and Supplementary Fig. 5 were averaged from multiple regions with a homemade MATLAB code to reduce noise. The plots were drawn using Origin 2018.

### STEM simulation

STEM simulation was carried out by Kirkland with COMPUTEM software^50^. Specifically, the technique involves dividing the sample into a number of thin slices normal to the incident electron beam and calculating the contribution to the cross-section at each slice. First, we used Vesta software to construct a 15 × 15 × 5 supercell containing 12,352 atoms based on the PbI_2_ CIF file^39^ and a 5 × 5 × 5 supercell containing 41,387 atoms from the MAPbI_3_ CIF file^51^. Then a homemade MATLAB code was used to rotate the structure to the desired zone axes. The STEM simulated parameters were set with the beam energy of 80 keV (accelerating voltage), object aperture of 25.1 mrad (convergence semi-angle), and a STEM ADF detector of 53–260 mrad (collection semi-angle). Besides, the transmission function size was 2048 × 2048 pixels and STEM probe size was 512 × 512 pixels. The other parameters were set as default. To achieve realistic computing time for these configurations, a limited area was chosen to run the STEM simulations. The thickness of the sample along different zone axes was estimated to range from 4.5 to 6.5 nm.

### Ab initio simulation

All ab initio simulations were performed using Vienna Ab Initio Simulation Package^52–54^ projected augmented wave (PAW)^55,56^ potential. For calculations of PbI_2_, strongly constrained and appropriately normed (SCAN)^57^ exchange-correlation functional was adopted. Plane-wave cutoff of 650 eV was used. For structural optimization, 6 × 6 × 6 k-mesh was used. For molecular dynamics simulations, 1 × 1 × 1 k-mesh and 81-atom supercell were used. Isothermal–isobaric ensemble (NPT)^58^ simulations at 300 K were run 5000 steps to obtain averaged lattice constants and subsequently, canonical ensemble (NVT) simulations at the same temperature were run 10,000 steps using Nosé–Hoover thermostat^59,60^, of which the final 8000 steps were used to average the atomic positions. The time step was chosen to be 2 fs for both simulations and the energy drift was less than 1 meV (ps)^−1^ per atom for NVT simulations.

## Electronic supplementary material

Supplementary Information

## Data Availability

The authors declare that all relevant data are included in the paper and its Supplementary Information files. Additional data including the codes are available from the corresponding author upon reasonable request.
