# Supplementary material for: Atomic scale insights into structure instability and decomposition pathway of methylammonium lead iodide perovskite
Source: Nat Commun. 2018 Nov 15;9:4807. doi: 10.1038/s41467-018-07177-y (PMC6237850; doi:10.1038/s41467-018-07177-y)
Supplement: Supplementary file 1 — Supplementary Information [file 41467_2018_7177_MOESM1_ESM.pdf]

Supplementary Information for

Atomic scale insights into structure instability and decomposition pathway of methylammonium lead iodide perovskite

Chen *et al.*

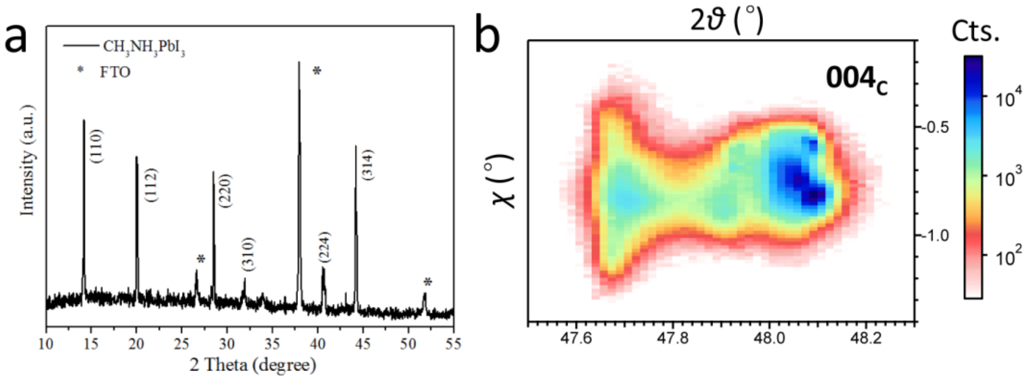

Supplementary Figure 1 | Single crystalline of tetragonal $CH_3NH_3PbI_3$ ($MAPbI_3$). **a** The powder X-ray diffraction (XRD). **b** The selected reflection from synchrotron XRD.

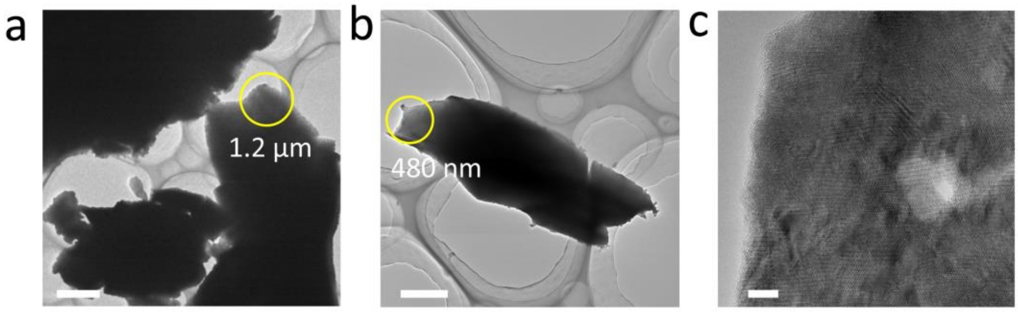

Supplementary Figure 2 | Transmission electron microscopy (TEM) images of tetragonal $MAPbI_3$. **a**, **b** TEM images used for selected area electron diffraction (SAED) pattern corresponding to Fig. 1d and Fig. 1h. Yellow circles mark the position of the selected area to record the electron diffraction (ED) pattern. **c** A high resolution TEM (HRTEM) image for fast Fourier transform (FFT) pattern in Fig. 1f. Scale bar, 1 µm (**a**); 500 nm (**b**); 10 nm (**c**).

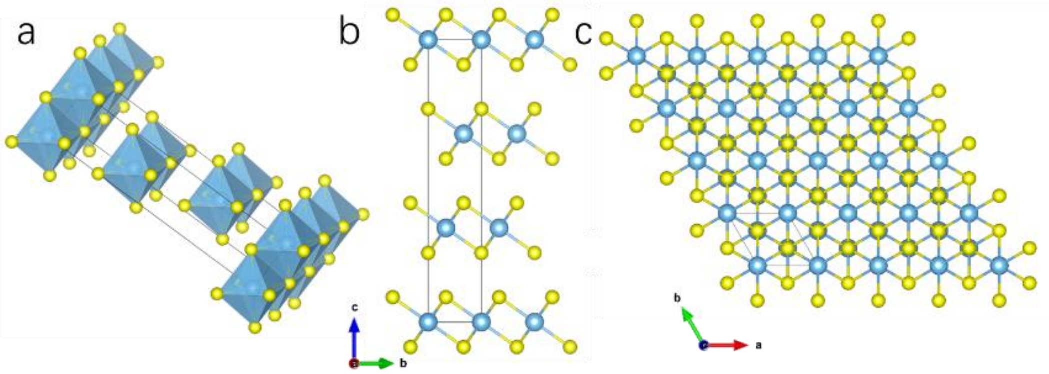

Supplementary Figure 3 | The atomistic configurations of hexagonal PbI_2. **a** The Pb-I_6 octahedra in PbI_2. **b**, **c** The ball and stick models along **b** the [100] and **c** [001] directions.

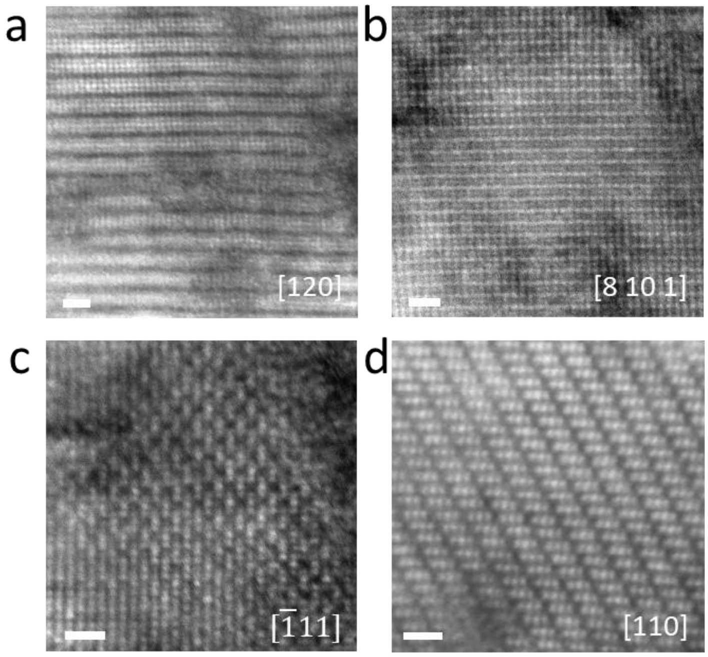

Supplementary Figure 4 | Atomic-resolution high angle annular dark field scanning transmission electron microscopy (HAADF STEM) images of PbI_2 with different viewing directions. **a-d** These are original STEM images without any post processing. Scale bar, 1 nm (**a-d**).

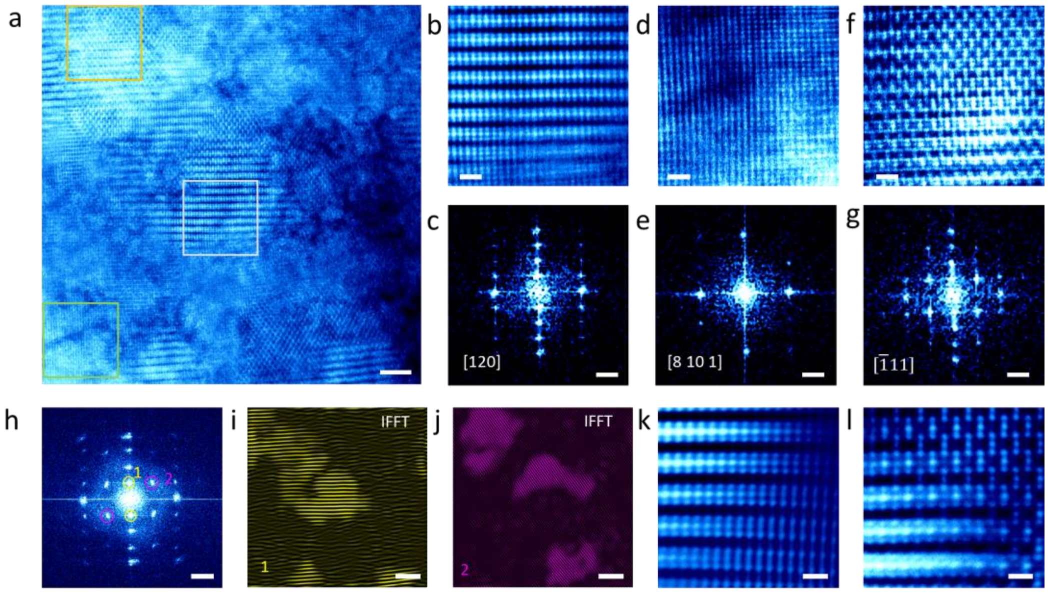

Supplementary Figure 5 | The co-existence of PbI_2 along different zone axes. **a** A STEM image of PbI_2. **b**, **d**, **f** Enlarged views of PbI_2 from the regions in **a** highlighted by **b** gray, **d** green, and **f** orange rectangles. **c**, **e**, **g** The corresponding FFT patterns along [120], [8 10 1] and [$\bar{1}$11] directions of PbI_2. **h** The FFT pattern of **a**. **i**, **j** The inverse FFT images using spots marked by 1 for **i** and 2 for **j**, showing the spatial distribution of PbI_2 along different directions. **k**, **l** The interfaces between different grains. Scale bar, 3 nm (**a**); 1 nm (**b**, **d**, **f**); 2 nm^{-1} (**c**, **e**, **g**, **h**); 5 nm (**i**, **j**); 0.5 nm (**k**, **l**).

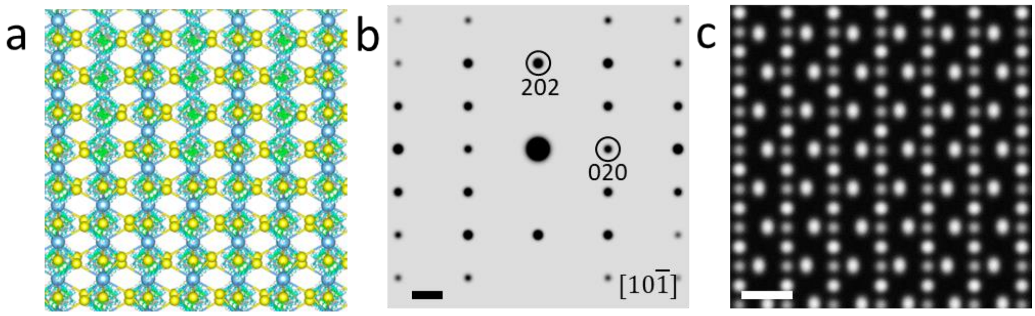

Supplementary Figure 6 | Ball and stick model, simulated ED pattern and STEM image of tetragonal $MAPbI_3$. **a** The atomic ball and stick model with **b** the simulated ED pattern and **c** simulated STEM image along the [10$\bar{1}$] zone axis. Scale bar, 0.5 nm^{-1} (**b**); 0.5 nm (**c**).

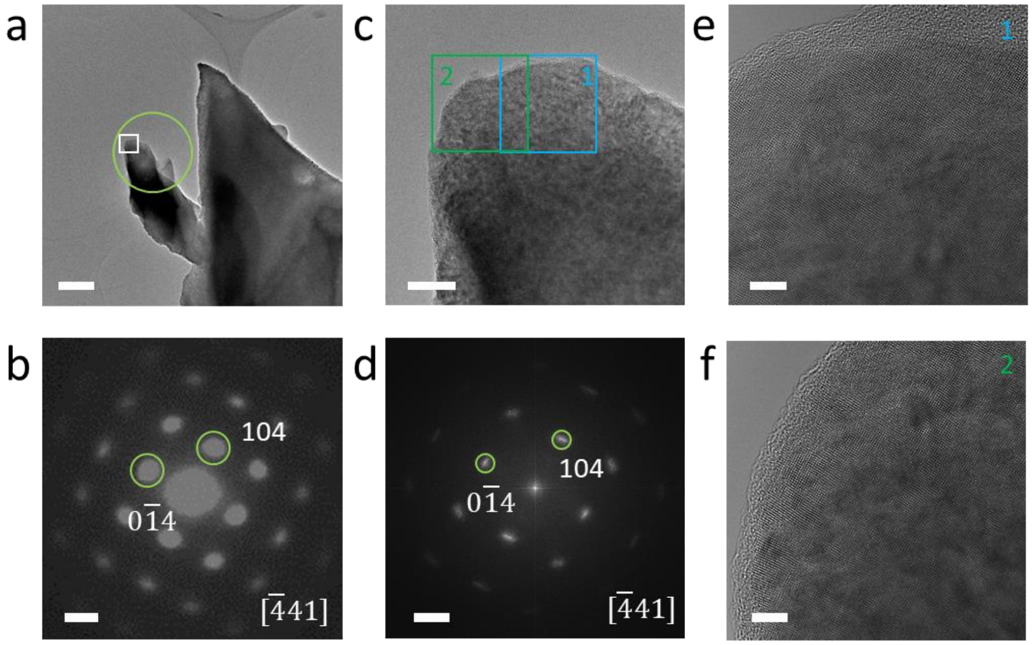

Supplementary Figure 7 | TEM images, SAED and FFT patterns of PbI_2 along the $[\bar{4}41]$ zone axis. **a** A TEM image and **b** the corresponding SAED pattern. The green circle in **a** shows the selected area to record the ED pattern. **c** A HRTEM image of the white square in **a** and the corresponding **d** FFT pattern. **e**, **f** HRTEM images of enlarged areas in **c** marked as blue and green squares. Scale bar, 200 nm (**a**); 2 nm^{-1} (**b**, **d**); 20 nm (**c**); 5 nm (**e**, **f**).

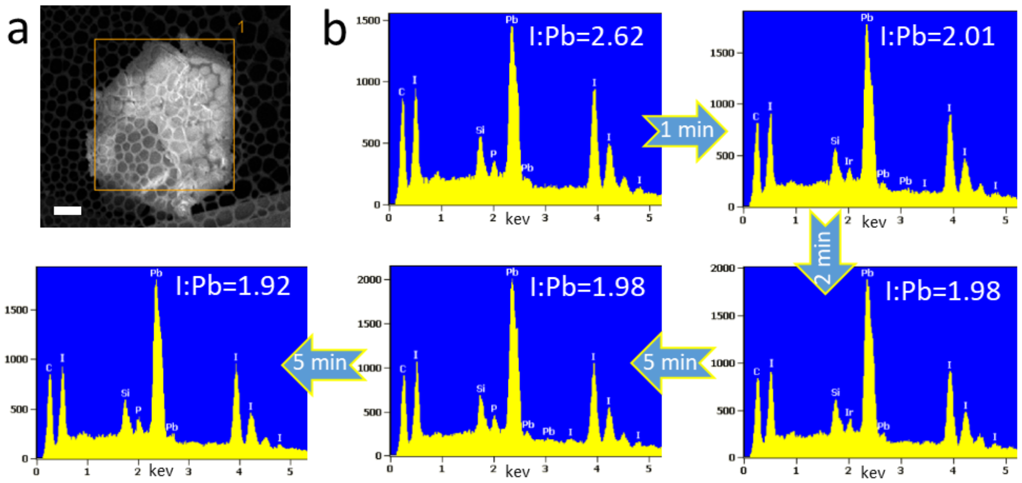

Supplementary Figure 8 | The quantitative EDX analysis during the degradation of $MAPbI_3$. **a** A SEM image of the sample. **b** The corresponding spectra obtained in **a** marked as an orange square. The beam was blank for certain time as shown on the arrows after each spectrum was acquired. Scale bar, 10 µm (**a**).

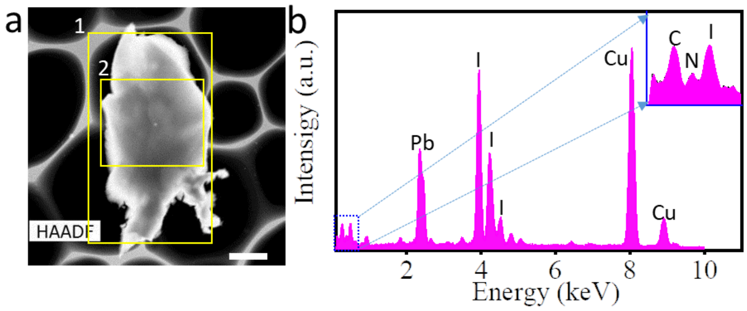

Supplementary Figure 9 | STEM-EDX analysis. **a** A STEM image of the PbI_2 and **b** the corresponding spectrum. Scale bar, 500 nm (**a**).

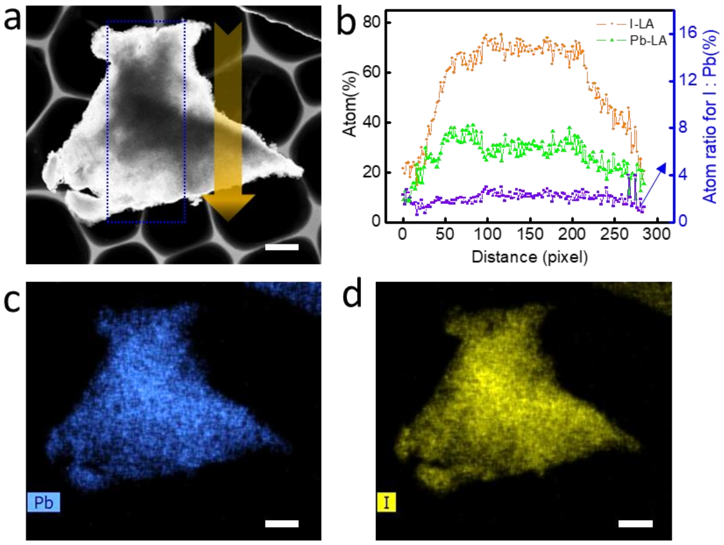

Supplementary Figure 10 | EDX mappings analysis. **a** A STEM image of PbI_2. **b** The quantitative result of the atom ratio acquired from the blue rectangle area in **a** with an average atom proportion ~2 for I/Pb. The orange arrow in **a** shows the direction of the line profile. **c**, **d** The corresponding EDX mappings of Pb and I. Scale bar, 1 μm (**a**, **c**, **d**).

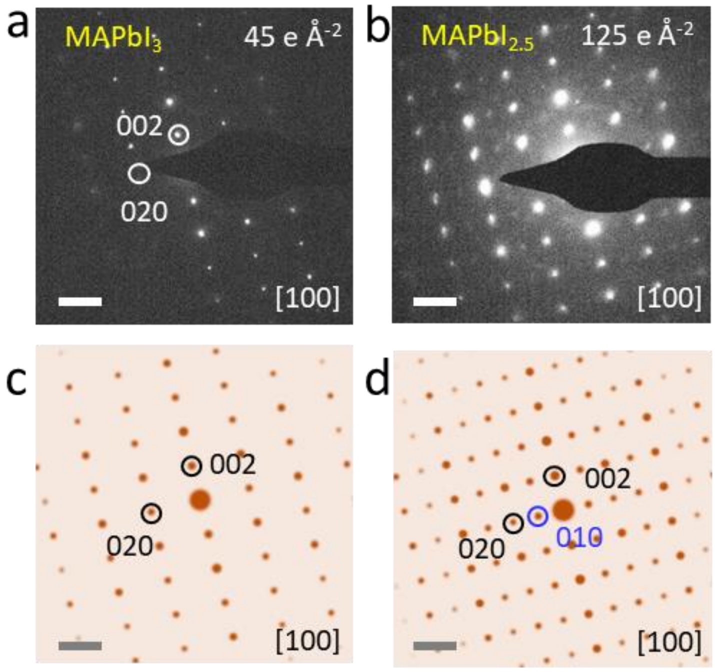

Supplementary Figure 11 | The SAED and simulated ED patterns of $MAPbI_3$ and $CH_3NH_3PbI_{2.5}$ ($MAPbI_{2.5}$). The SAED patterns of **a** $MAPbI_3$ and **b** $MAPbI_{2.5}$ along the [100] zone axis. **c**, **d** The corresponding simulated ED patterns. The dose rate is ~1.5 e $Å^{-2}$ s^{-1}. Scale bar, 1 nm^{-1} (**a**-**d**).

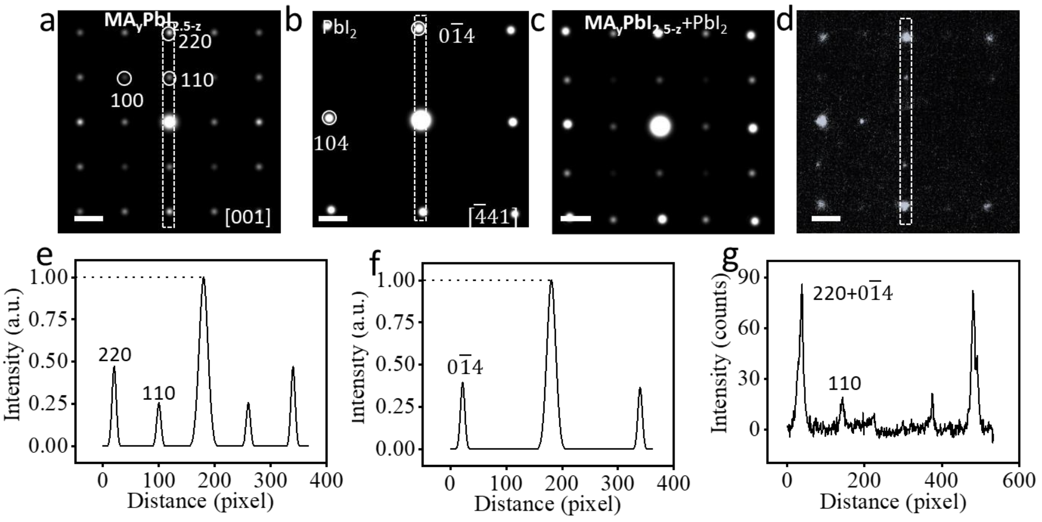

Supplementary Figure 12 | The co-existence of $MA_yPbI_{2.5\text{-}z}$ and PbI_2. Simulated ED patterns along **a** the $MA_yPbI_{2.5\text{-}z}$ (y=0 and z=0.5) [001] direction and **b** PbI_2 [$\bar{4}$41] direction and **c** the overlap of **a** and **b**. Note that $MA_yPbI_{2.5\text{-}z}$ ($0\leqslant y\leqslant 1$ and $0\leqslant z\leqslant 0.5$) with random vacancies should have the same diffraction spots with $MA_yPbI_{2.5\text{-}z}$ (y=0 and z=0.5) without considering the very diffused reflections from random vacancies. **d** The experimental ED pattern. **e-g** The corresponding line-profiles obtained along the dashed white rectangles in **a**, **b**, **d**. Note that the intensities of (000) spots are normalized to be one as shown in **e** and **f**. According to the experimental and normalized simulated intensities, the proportions of $MA_yPbI_{2.5\text{-}z}$ and PbI_2 are ~65.0% and ~35.0%, respectively. Scale bar, 1 nm^{-1} (**a-d**)

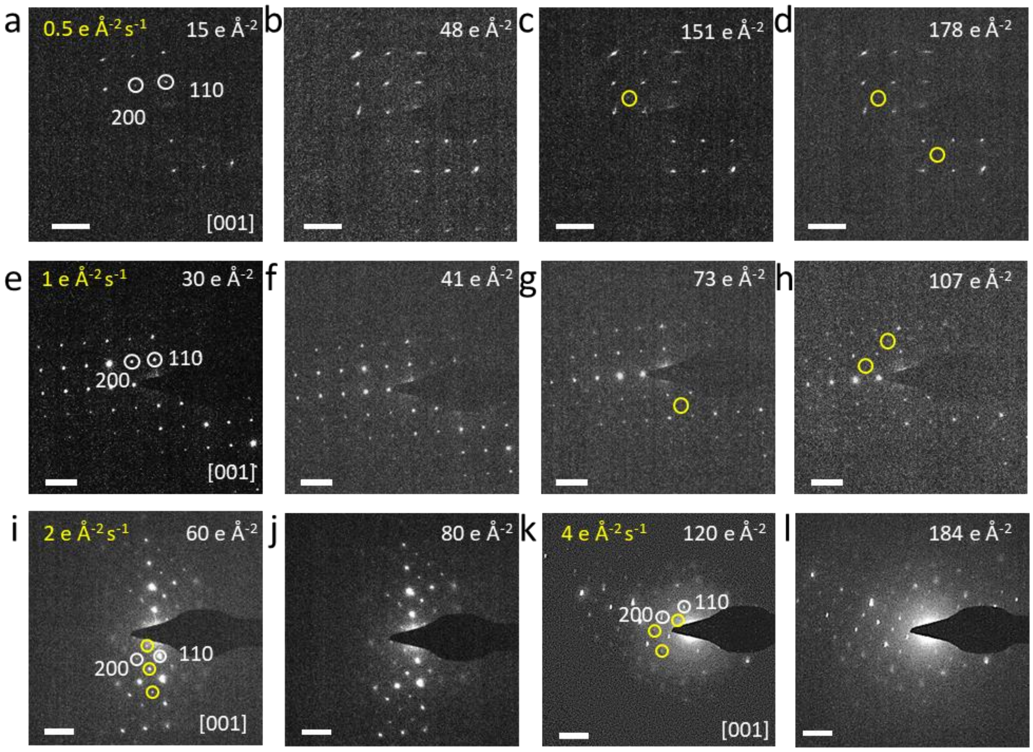

Supplementary Figure 13 | The structure evolution at different dose rates. **a-d** At a dose rate of 0.5 e Å^{-2} s^{-1}, the superstructure reflections were first observed at the total dose of 151 e Å^{-2}. **e-h** At a dose rate of 1 e Å^{-2} s^{-1}, the superstructure reflections were first observed at the total dose of 73 e Å^{-2}. At **i**, **j** 2 e Å^{-2} s^{-1} and **k**, **l** 4 e Å^{-2} s^{-1}, the superstructure was observed in the first recorded ED images. Note that it took about 30 s to acquire SAED image after the sample came into sight. The yellow circles highlight the superstructure reflections. Scale bar, 2 nm^{-1} (**a-l**)

Supplementary Table 1 A comprehensive survey of literatures reporting on ED and FFT patterns of tetragonal $MAPbI_3$

Zone axis	Diffraction spots		Details or methods	Reference
[001]	(110) (×)	(1-10) (×)	FFT	Nano Energy 2017 (ref. 1)
[001]	(110) (×)	(1-10) (×)	SAED	Sci. Bull. 2016 (ref. 2)
[001]	(110) (×)	(1-10) (×)	SAED	Nano Lett. 2016 (ref. 3)
[001]	(110) (×)	(1-10) (×)	SAED	Nat. Energy 2016 (ref. 4)
[201]	(11-2) (×)	(-112) (×)	SAED	Nat. Energy 2016 (ref. 4)
[001]	(110) (×)	(1-10) (×)	SAED	Nat. Commun. 2016 (ref. 5)
[001]	(110) (×)	(1-10) (×)	SAED	J. Phys. Chem. Lett 2015 (ref. 6)
[110]	(-110) (×)	(002) (×)	SAED and FFT	Nat. Mater. 2015 (ref. 7)
[110]	(-110) (×)	(002) (×)	FFT	ACS Nano 2015 (ref. 8)
[110]	(-110) (×)	(002) (×)	SAED and FFT	Angew. Chem. Int. Ed. 2015 (ref. 9)
[001]	(110) (×)	(1-10) (×)	SAED	Adv. Mater. 2015 (ref. 10)
[001]	(110) (×)	(1-10) (×)	SAED and FFT	Angew. Chem. Int. Ed. 2014 (ref. 11)
Unidentified	(110) (×)	(1-10) (×)	FFT	Sci. Rep. 2017 (ref. 12)
Unidentified	(110) (×)	(012) (×)	FFT	Mater. Res. Express 2014(ref. 13)
Unidentified	(110) (×)	(002) (×)	HRTEM	Angew. Chem. Int. Ed. 2014 (ref. 14)
[110]			Without scale bar and identification of spots	J. Mater. Chem. A 2016 (ref. 15)
Unidentified	(220) (√)	(110) (×)	SAED	Crys. Res. Technol. 2017 (ref. 16)
Unidentified	(20-2) (√)	(10-1) (×)	FFT	ChemPhysChem 2015 (ref. 17)
[1-10]	(110) (√)	(002) (√)	SAED, (110) and (1-10) spots are dimmer	Nano. Lett. 2015 (ref. 18)
[1-10]	(110) (√)	(002) (√)	SAED dose rate ~1e $Å^{-2}$ s^{-1}	Nat. Commun. 2017 (ref. 19)
[110]	(-110) (√)	(002) (√)	SAED	ACS Energy Lett. 2017 (ref. 20)
[001]	(110) (√)	(1-10) (√)	SAED	This work

Note that the [110] and [001] zone axes are equal for tetragonal $MAPbI_3$.
In table, the cross means the corresponding spots are absent while the tick indicates their existence.

Supplementary Table 2 The quantitative STEM-EDX results of PbI_2

Atomic-% (norm.)	C	N	Pb	I	I:Pb
Region 1	13.59	7.77	26.47	53.17	2
Region 2	10.27	7.42	27.19	55.01	2.02

The region 1 and region 2 are from Supplementary Fig. 9a marked as 1 and 2.

Supplementary References:

1. Textured $CH_3NH_3PbI_3$ thin film with enhanced stability for high performance perovskite solar cells. *Nano Energy* **33**, 485-496 (2017).
2. Zhao, C. et al. Diffusion-correlated local photoluminescence kinetics in $CH_3NH_3PbI_3$ perovskite single-crystalline particles. *Sci. Bull.* **61**, 665-669 (2016).
3. Gao, L. et al. Passivated single-crystalline $CH_3NH_3PbI_3$ nanowire photodetector with high detectivity and polarization sensitivity. *Nano Lett.* **16**, 7446-7454 (2016).
4. Self-formed grain boundary healing layer for highly efficient $CH_3NH_3PbI_3$ perovskite solar cells. *Nat. Energy* **1**, 16081 (2016).
5. Li, D. et al. Size-dependent phase transition in methylammonium lead iodide perovskite microplate crystals. *Nat. Commun.* **7**, 11330 (2016).
6. Zhou, Y. et al. Crystal morphologies of organolead trihalide in mesoscopic/planar perovskite solar cells. *J. Phys. Chem. Lett.* **6**, 2292-2297 (2015).
7. Zhu, H. et al. Lead halide perovskite nanowire lasers with low lasing thresholds and high quality factors. *Nat. Mater.* **14**, 636-642 (2015).
8. Zhu, F. et al. Shape evolution and single particle luminescence of organometal halide perovskite nanocrystals. *ACS Nano* **9**, 2948-2959 (2015).
9. Kollek, T. et al. Porous and shape-anisotropic single crystals of the semiconductor perovskite $CH_3NH_3PbI_3$ from a single-source precursor. *Angew. Chem. Int. Ed.* **54**, 1341-1346 (2015).
10. Yang, M. et al. Square-centimeter solution-processed planar $CH_3NH_3PbI_3$ perovskite solar cells with efficiency exceeding 15%. *Adv. Mater.* **27**, 6363-6370 (2015).
11. Xiao, M. et al. A fast deposition-crystallization procedure for highly efficient lead iodide perovskite thin-film solar cells. *Angew. Chem. Int. Ed.* **53**, 9898-9903 (2014).
12. Kim, S. et al., Relationship between ion migration and interfacial degradation of $CH_3NH_3PbI_3$ perovskite solar cells under thermal conditions. *Sci. Rep.* **7** 1200, (2017).

13. Zhenhua, C. A. H. L., Shape-controlled synthesis of organolead halide perovskite nanocrystals and their tunable optical absorption. *Mater. Res. Express* **1** 15034 (2014).
14. Xiao, M. et al. A fast deposition-crystallization procedure for highly efficient lead iodide perovskite thin-film solar cells. *Angew. Chem. Int. Ed.* **53**, 9898-9903 (2014).
15. Ye, T. et al., Single-crystalline lead halide perovskite arrays for solar cells. *J. Mater. Chem. A* **4** 1214 (2016).
16. Su, J. et al., Growth and properties of $CH_3NH_3PbI_3$ single crystal. *Crys. Res. Technol.* **52** 1700171 (2017).
17. Alberti, A. et al., Similar structural dynamics for the degradation of $CH_3NH_3PbI_3$ in air and in vacuum. *ChemPhysChem* **16** 3064 (2015).
18. Xing, J. et al., Vapor phase synthesis of organometal halide perovskite nanowires for tunable room-temperature nanolasers. *Nano. Lett.* **15** 4571 (2015).
19. Rothmann, M.U. et al. Direct observation of intrinsic twin domains in tetragonal $CH_3NH_3PbI_3$. *Nat. Commun.* **8**, 14547 (2017).
20. Ji, F. et al. Simultaneous evolution of uniaxially oriented grains and ultralow-density grain-boundary network in $CH_3NH_3PbI_3$ perovskite thin films mediated by precursor phase metastability. *ACS Energy Lett.* **2**, 2727-2733 (2017).
